# Supplementary material for: ALPL-1 is a target for chimeric antigen receptor therapy in osteosarcoma
Source: Nat Commun. 2023 Jun 8;14:3375. doi: 10.1038/s41467-023-39097-x (PMC10250459; doi:10.1038/s41467-023-39097-x)
Supplement: Supplementary file 1 — Supplementary Information [file 41467_2023_39097_MOESM1_ESM.pdf]

SUPPLEMENTARY INFORMATION

ALPL-1 is a target for chimeric antigen receptor therapy in osteosarcoma

Nadia Mensali[1], Hakan Köksal[1,20], Sandy Joaquina[1,20], Patrik Wernhoff[1], Nicholas P. Casey[1], Paola Romecin[2,3], Carla Panisello[2,3], René Rodriguez[4,5,6], Lene Vimeux[7], Asta Juzeniene[8], Marit R. Myhre[1], Anne Fåne[1], Carolina Castilla Ramírez[9], Solrun Melkorka Maggadottir[1], Adil Doganay Duru[10], Anna-Maria Georgoudaki[10,11], Iwona Grad[8], Andrés Daniel Maturana[12], Gustav Gaudernack[13], Gunnar Kvalheim[1], Angel M. Carcaboso[14], Enrique de Alava[9,15], Emmanuel Donnadieu[7], Øyvind S. Bruland[16], Pablo Menendez[2,3,17,18,19], Else Marit Inderberg[1, ✉] &Sébastien Wälchli[1, ✉]

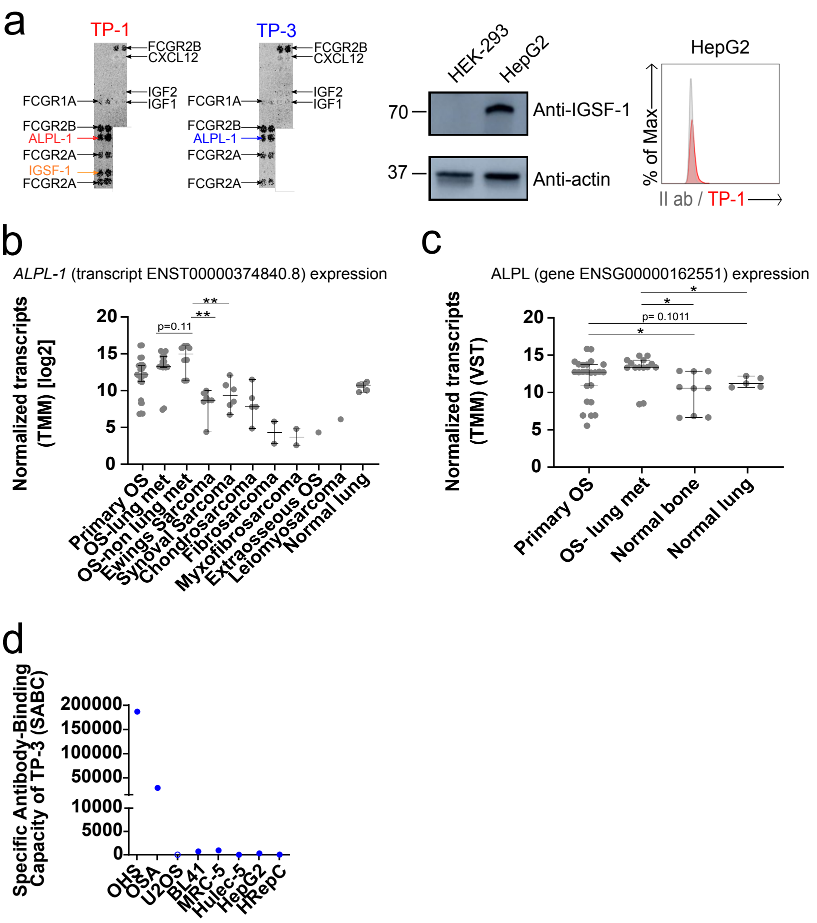

Supplementary Figure 1. TP antibody specificity and affinity. **a,** Target identification and cross-reactivity screening of TP-1 and TP -3 antibodies using Retrogenix screening technology (left, was repeated two times); Western blotting showing presence of IGSF -1 proteins in Hep G2 (middle) (n=2, uncropped Western blotting in supplementary information); flow cytometric histograms showing staining of HepG2 with TP-1 antibodies (right). b, Expression of ALPL-1 transcript ENST00000374840.8 in sarcomas (Primary OS n= 24, OS -lung met astastic (met) n=14, OS -non lung met n= 8, Ewings Sarcoma n=7, Synoval Sarcoma n= 6, Chondrosarcoma n=5, Fibrosarcoma n=2, Myxofibrosarcoma n= 2, Extraosseous OS n=1, Leiomyosarcoma n=1) and normal lung (n=5), (met, metastasis). The expression data is normalized by Trimmed mean of M values (TMM) method prior to log2 transformation . Statistical comparisons were performed with two-tailed Man-Whitney test ns, not significant p>0.05, *p<0.05, **p<0.01). **c**, Expression of *ALPL* gene ENSG00000162551 in primary (n=24) and metastatic (met) OS (n=14) and in normal tissues for bone (n=9) and lung (n=5). Normalized expression data presented using VST (variance stabilizing transformation). Statistical comparisons were performed with two-tailed Mann-Whitney test ns, not significant p>0.05, *p<0.05). **b,c,** Data are presented as median with 95% confidence intervalley. **d**, Specific antibody binding capacity of TP -3 against OS cell lines (OHS, OSA and U2OS), lymphoma (BL-41), healthy tissue cells representing lung (MRC-5, Hulec-5a, HPAEpiC), liver (HH), and kidney (HREpC).

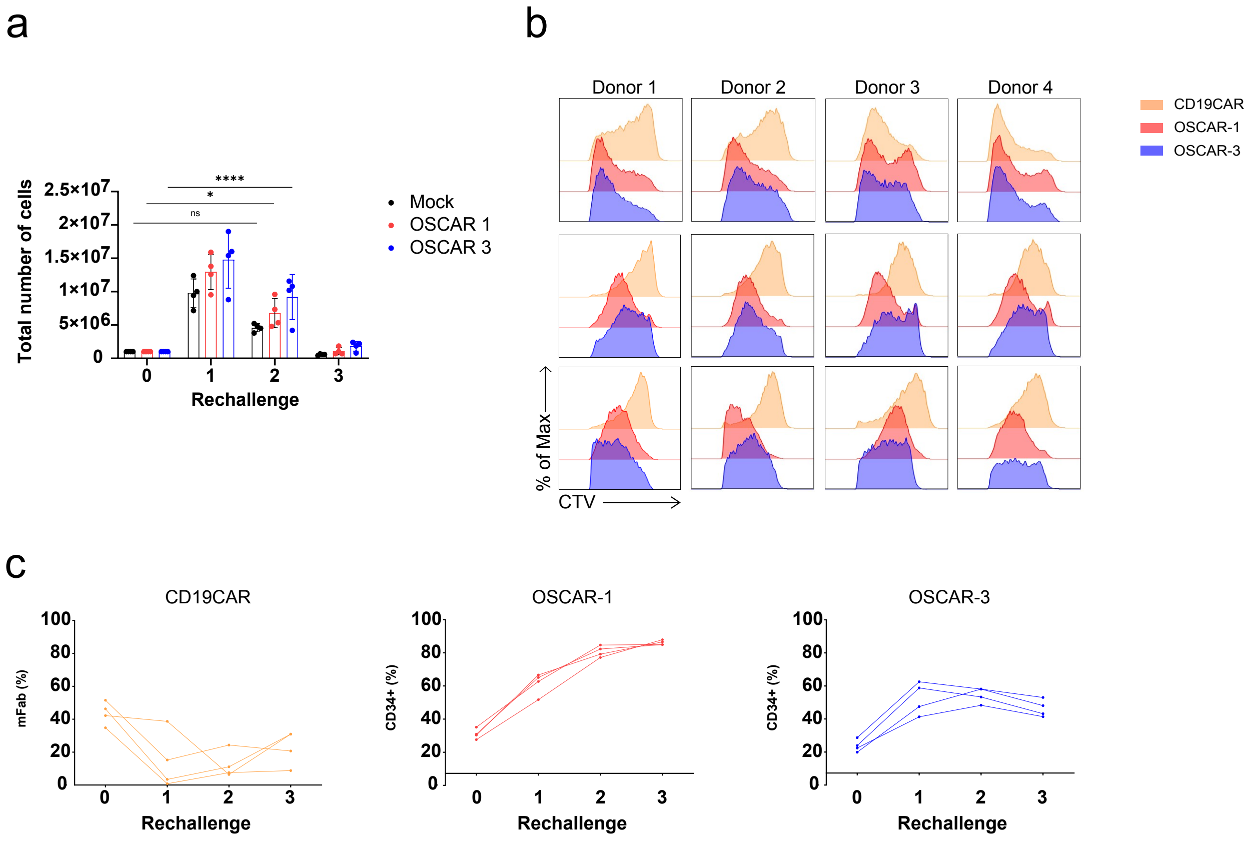

Supplementary Figure 2. a, Proliferation capability of OSCAR T cells upon rechallenge with OHS cell line. Data are shown as independent values of four donors (n=4). Two-way ANOVA with Tukey's multiple comparison test was used to calculate p values (ns, not significant p>0.05, *p<0.05, ****p<0.0001). **b**, Histograms of CTV staining of CAR T cells after 1st (top panel), 2nd (middle panel) and 3rd (bottom panel) antigen stimulation (HEK-293 $^{ALPL-1}$). Mean fluorescence intensity (MFI) of CTV in CD19CAR (orange), OSCAR-1 (red) and OSCAR-3 (blue) is shown in four donors. **c,** Monitoring of CAR expression after repeated antigen stimulation with HEK-293^{ALPL-1} (three rechallenges). OSCAR-1 and 3 were detected by CD34 staining, and CD19CAR by mFab staining. Data are shown as independent values of four donors (n=4).

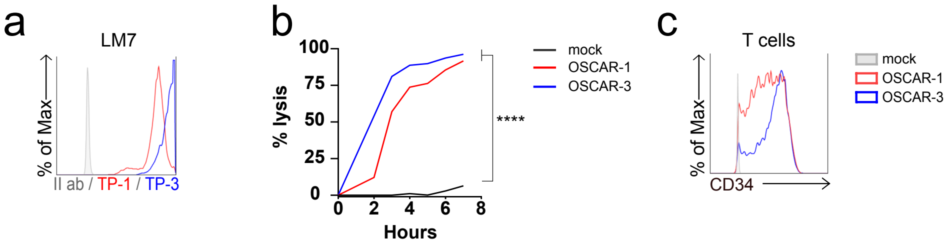

Supplementary Figure 3. *In vitro* effector function of OSCAR-1 and OSCAR-3 T cells towards LM7 OS cells. **a**, Representative flow cytometric histograms showing staining of LM7 OS cells with TP-1 and TP-3 antibodies. **b**, Kinetics of *in vitro* cytotoxicity of OSCAR-1 and OSCAR-3 T cells against LM7 cells at an E:T ratio of 10:1 (in red and blue). Lysis of LM7 cells is monitored up to 7 hours. Data represents mean ± s.d. of two independent experiments (n=2). Statistical comparisons are performed with two-way ANOVA with Tukey's multiple comparison test (ns, not significant $p>0.05$, **** $p<0.0001$).
c, Expression level of OSCAR-1 and OSCAR-3 CAR in T cells used in the cytotoxicity assay.

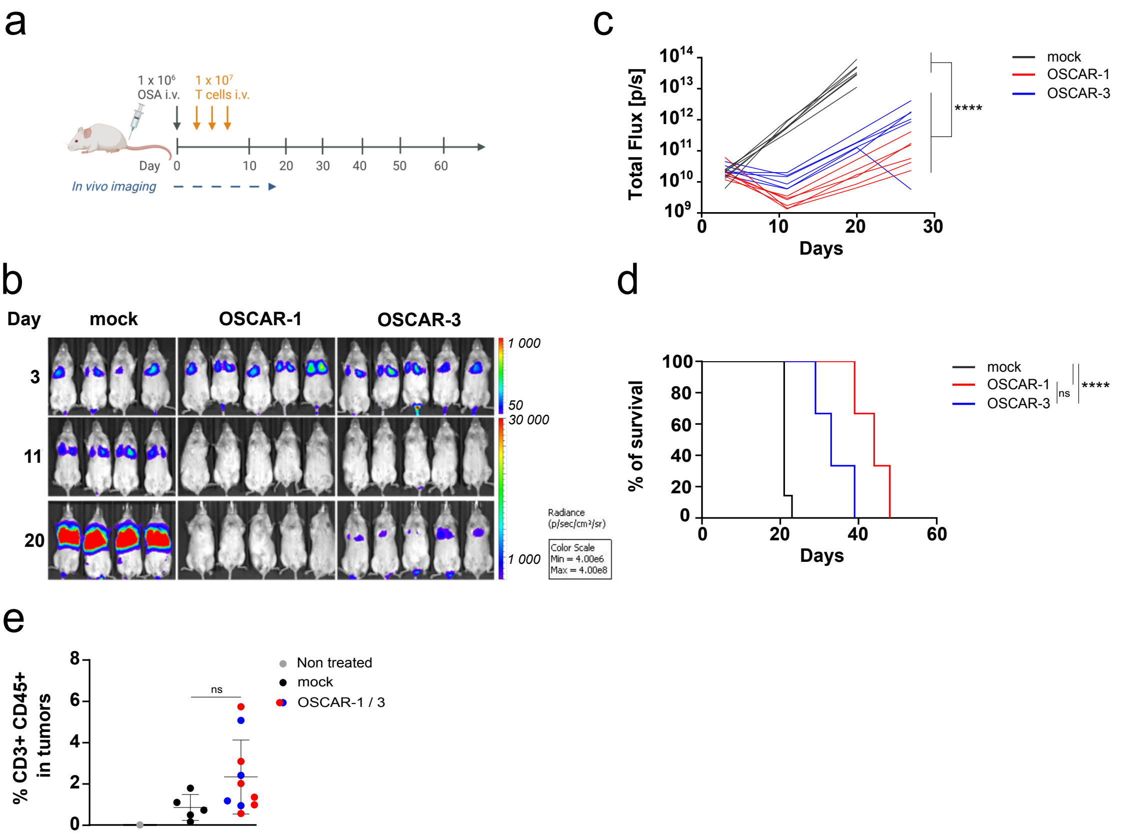

Supplementary Figure 4. ***In vivo*** **effector function of OSCAR-1 and OSCAR-3 T cells in lung metastatic mouse models.** **a**, Experimental overview of OSA OS lung model. NSG mice were engrafted with GFP/Luc+ OSA cells i.v. On day 10, mice were randomized and received 3 i.v. injections of mock, OSCAR-1, or OSCAR-3 T cells. **b**, Tumor burden measured by IVIS. Representative BLI images of the luminescent signal from each treatment group. **c**, Quantification of tumor progression for each individual mouse per each group measured by flux values acquired via BLI. Statistical comparisons were performed with two-way ANOVA with Tukey's multiple comparison test (ns, not significant ****$p<0.0001$). **d**, Kaplan-Meier survival curves using a Mante -Cox (log-rank) test (ns, not significant $p>0.05$, ****$p<0.0001$). Experiment was performed once; five mice per group were included (n=5). **e**, T cell homing experiment showing percentage of CD3+/CD45+ cells found in tumor tissues from lungs. 3 mice per treatment group were injected i.v. with 1×10^6 GFP/Luc+ OSA cells. Tumor-bearing mice were randomized and injected once with 1×10^7 mock, OSCAR-1, or OSCAR-3 T cells. Mice were euthanized 4 days after T cell injection and lungs were dissected. Data are presented as mean ± s.d. Statistical comparisons were performed with two-tailed unpaired Student t-test (ns, not significant $p= 0.1849$).

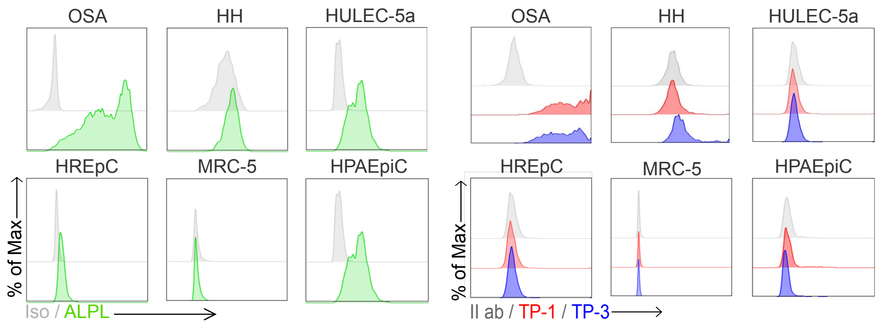

Supplementary Figure. 5 Staining of target cells in figure 5b with anti ALPL, TP-1 and TP-3 antibodies. Isotype and secondary antibody controls (grey).

Sample Id	Type	Sample Alias	Condition	Source
10_S1	Synovial sarcoma	HTT10	Tumor	NSUC
12_S2	Extraoseos osteosarcoma	HTT12	Tumor	NSUC
16_S3	Pleomorphic myxofibrosarcoma	HTT16	Tumor	NSUC
17_S4	Primary OS	HTT17	Tumor	NSUC
21_S5	Myxofibrosarcoma	HTT21	Tumor	NSUC
25_S6	Pleaomorphic spindle cell sarcoma	HTT25	Tumor	NSUC
26_S7	Chondrosarcoma	HTT26	Tumor	NSUC
29_S8	Chondrosarcoma	HTT29	Tumor	NSUC
30_S1	Chondrosarcoma	HTT30	Tumor	NSUC
31_S2	High grade pleomorphic fibrosarcoma	HTT31	Tumor	NSUC
38_S3	ST_sarcoma	HTT38	Tumor	NSUC
39_S4	Primary OS	HTT39	Tumor	NSUC
41_S5	Fibrosarcoma	HTT41	Tumor	NSUC
42_S6	Fibrosarcoma	HTT42	Tumor	NSUC
47_S7	Ewings sarcoma	HTT47	Tumor	NSUC
52_S8	Myxofibrosarcoma	HTT52	Tumor	NSUC
58_S1	Leiomyosarcoma	HTT58	Tumor	NSUC
62_S2	Chondrosarcoma	HTT62	Tumor	NSUC
74_S3	n.d	HTT74	Tumor	NSUC
75_S4	n.d	HTT75	Tumor	NSUC
78_S5	Primary OS	HTT78	Tumor	NSUC
79_S6	Chondrosarcoma	HTT79	Tumor	NSUC
82_S7	Primary OS	HTT82	Tumor	NSUC
SRR10691631	Normal kidney	GSM4217781	Normal	ENA
SRR10691632	Normal kidney	GSM4217782	Normal	ENA
SRR10691633	Normal kidney	GSM4217783	Normal	ENA
SRR10691634	Normal kidney	GSM4217784	Normal	ENA
SRR10691635	Normal kidney	GSM4217785	Normal	ENA
SRR11804653	Ewings sarcoma	GSM4557259	Tumor	ENA
SRR11804654	Ewings sarcoma	GSM4557260	Tumor	ENA
SRR11804655	Ewings sarcoma	GSM4557261	Tumor	ENA
SRR11804656	Ewings sarcoma	GSM4557262	Tumor	ENA
SRR11804657	Ewings sarcoma	GSM4557263	Tumor	ENA
SRR11804658	Ewings sarcoma	GSM4557264	Tumor	ENA
SRR1265495	Normal lung	GSM1375986	Normal	ENA
SRR1265496	Normal lung	GSM1375987	Normal	ENA
SRR1265497	Normal lung	GSM1375988	Normal	ENA
SRR1265498	Normal lung	GSM1375989	Normal	ENA
SRR1265499	Normal lung	GSM1375990	Normal	ENA
SRR2748125	Highly-met OS cell line	GSM1915018	Tumor	ENA
SRR2748126	Highly-met OS cell line	GSM1915019	Tumor	ENA
SRR2748127	Highly-met OS cell line	GSM1915020	Tumor	ENA
SRR2748128	Highly-met OS cell line	GSM1915021	Tumor	ENA
SRR2748129	Highly-met OS cell line	GSM1915022	Tumor	ENA
SRR2748130	Non-met OS cell line	GSM1915023	Tumor	ENA
SRR2748131	Non-met OS cell line	GSM1915024	Tumor	ENA
SRR2748132	Highly-met OS cell line	GSM1915025	Tumor	ENA
SRR4343885	OS-lung met	HO002M	Tumor	ENA
SRR4343886	OS-lung met	HO002M	Tumor	ENA
SRR4343887	OS-lung met	HO002M	Tumor	ENA
SRR4343888	OS-lung met	HO002M	Tumor	ENA
SRR4343889	OS-lung met	HO002M	Tumor	ENA
SRR4343890	OS-lung met	HO002M	Tumor	ENA
SRR4343891	Primary OS	HO003	Tumor	ENA
SRR4343892	Primary OS	HO003	Tumor	ENA
SRR4343893	Primary OS	HO003	Tumor	ENA
SRR4343894	Primary OS	HO003	Tumor	ENA
SRR4343895	Primary OS	HO003	Tumor	ENA

Supplementary Table 1

Sample Id	Type	Sample Alias	Condition	Source
SRR4343896	Primary OS	HO003	Tumor	ENA
SRR4343897	Primary OS	HO004	Tumor	ENA
SRR4343898	Primary OS	HO004	Tumor	ENA
SRR4343899	Primary OS	HO004	Tumor	ENA
SRR4343900	Primary OS	HO004	Tumor	ENA
SRR4344033	OS-lung met	HO043M	Tumor	ENA
SRR4344034	OS-lung met	HO043M	Tumor	ENA
SRR4344035	OS-lung met	HO044M	Tumor	ENA
SRR4344036	OS-lung met	HO044M	Tumor	ENA
SRR4344039	OS-met	HO046M-ch	Tumor	ENA
SRR4344040	OS-met	HO046M-ch	Tumor	ENA
SRR4344041	OS-met	HO047M-ch	Tumor	ENA
SRR4344042	OS-met	HO047M-ch	Tumor	ENA
SRR4344043	OS-lung met	HO052M	Tumor	ENA
SRR4344044	OS-lung met	HO052M	Tumor	ENA
SRR4344045	OS-met	HO055-ch	Tumor	ENA
SRR4344046	OS-met	HO055-ch	Tumor	ENA
SRR4344047	OS-met	HO056M-U	Tumor	ENA
SRR4344048	OS-met	HO056M-U	Tumor	ENA
SRR4344049	Primary OS	HO057	Tumor	ENA
SRR4344050	Primary OS	HO057	Tumor	ENA
SRR4344051	OS-lung met	HO058M	Tumor	ENA
SRR4344052	OS-lung met	HO058M	Tumor	ENA
SRR4344053	Primary OS	HO059	Tumor	ENA
SRR4344054	Primary OS	HO059	Tumor	ENA
SRR4344055	Primary OS	HO060	Tumor	ENA
SRR4344056	Primary OS	HO060	Tumor	ENA
SRR4344057	Primary OS	HO062	Tumor	ENA
SRR4344058	Primary OS	HO062	Tumor	ENA
SRR4344059	Primary OS	HO063	Tumor	ENA
SRR4344060	Primary OS	HO063	Tumor	ENA
SRR4344073	Normal bone	HO069B	Normal	ENA
SRR4344074	Normal bone	HO069B	Normal	ENA
SRR4344075	Normal bone	HO069B	Normal	ENA
SRR4344076	Normal bone	HO070B	Normal	ENA
SRR4344077	Normal bone	HO070B	Normal	ENA
SRR4344078	Normal bone	HO070B	Normal	ENA
SRR4344079	Normal bone	HO071B	Normal	ENA
SRR4344080	Normal bone	HO071B	Normal	ENA
SRR4344081	Normal bone	HO071B	Normal	ENA
SRR5576263	Normal liver	hiPSC-hepatocyte	Normal	ENA
SRR5576264	Normal liver	HepaRGTM	Normal	ENA
SRR5576266	Normal liver	hESC-hepatocyte	Normal	ENA
SRR6373111	Synovial sarcoma	GSM2887701	Tumor	ENA
SRR6373112	Synovial sarcoma	GSM2887702	Tumor	ENA
SRR6373113	Synovial sarcoma	GSM2887703	Tumor	ENA
SRR6373114	Synovial sarcoma	GSM2887704	Tumor	ENA
SRR6373115	Synovial sarcoma	GSM2887705	Tumor	ENA
SRR6373116	Synovial sarcoma	GSM2887706	Tumor	ENA
Undetermined_S0	n.d	n.d	Tumor	NSUC

Supplementary Table 1

Sample Id	Type	Sample Name	Condition	Source
17_S4	Primary OS	HTT17	Tumor	NSUC
39_S4	Primary OS	HTT39	Tumor	NSUC
78_S5	Primary OS	HTT78	Tumor	NSUC
82_S7	Primary OS	HTT82	Tumor	NSUC
SRR1265495	Normal lung	GSM1375986	Normal	ENA
SRR1265496	Normal lung	GSM1375987	Normal	ENA
SRR1265497	Normal lung	GSM1375988	Normal	ENA
SRR1265498	Normal lung	GSM1375989	Normal	ENA
SRR1265499	Normal lung	GSM1375990	Normal	ENA
SRR4343885	OS-lung met	HO002M	Tumor	ENA
SRR4343886	OS-lung met	HO002M	Tumor	ENA
SRR4343887	OS-lung met	HO002M	Tumor	ENA
SRR4343888	OS-lung met	HO002M	Tumor	ENA
SRR4343889	OS-lung met	HO002M	Tumor	ENA
SRR4343890	OS-lung met	HO002M	Tumor	ENA
SRR4343891	Primary OS	HO003	Tumor	ENA
SRR4343892	Primary OS	HO003	Tumor	ENA
SRR4343893	Primary OS	HO003	Tumor	ENA
SRR4343894	Primary OS	HO003	Tumor	ENA
SRR4343895	Primary OS	HO003	Tumor	ENA
SRR4343896	Primary OS	HO003	Tumor	ENA
SRR4343897	Primary OS	HO004	Tumor	ENA
SRR4343898	Primary OS	HO004	Tumor	ENA
SRR4343899	Primary OS	HO004	Tumor	ENA
SRR4343900	Primary OS	HO004	Tumor	ENA
SRR4344033	OS-lung met	HO043M	Tumor	ENA
SRR4344034	OS-lung met	HO043M	Tumor	ENA
SRR4344035	OS-lung met	HO044M	Tumor	ENA
SRR4344036	OS-lung met	HO044M	Tumor	ENA
SRR4344043	OS-lung met	HO052M	Tumor	ENA
SRR4344044	OS-lung met	HO052M	Tumor	ENA
SRR4344049	Primary OS	HO057	Tumor	ENA
SRR4344050	Primary OS	HO057	Tumor	ENA
SRR4344051	OS-lung met	HO058M	Tumor	ENA
SRR4344052	OS-lung met	HO058M	Tumor	ENA
SRR4344053	Primary OS	HO059	Tumor	ENA
SRR4344054	Primary OS	HO059	Tumor	ENA
SRR4344055	Primary OS	HO060	Tumor	ENA
SRR4344056	Primary OS	HO060	Tumor	ENA
SRR4344057	Primary OS	HO062	Tumor	ENA
SRR4344058	Primary OS	HO062	Tumor	ENA
SRR4344059	Primary OS	HO063	Tumor	ENA
SRR4344060	Primary OS	HO063	Tumor	ENA
SRR4344073	Normal bone	HO069B	Normal	ENA
SRR4344074	Normal bone	HO069B	Normal	ENA
SRR4344075	Normal bone	HO069B	Normal	ENA
SRR4344076	Normal bone	HO070B	Normal	ENA
SRR4344077	Normal bone	HO070B	Normal	ENA
SRR4344078	Normal bone	HO070B	Normal	ENA
SRR4344079	Normal bone	HO071B	Normal	ENA
SRR4344080	Normal bone	HO071B	Normal	ENA
SRR4344081	Normal bone	HO071B	Normal	ENA

Note:

n.d = Not determined, NSUC = Nova Southeastern University Center, ENA = European Nucleotide Archive.

Supplementary Table 1

SUPPLEMENTARY METHODS

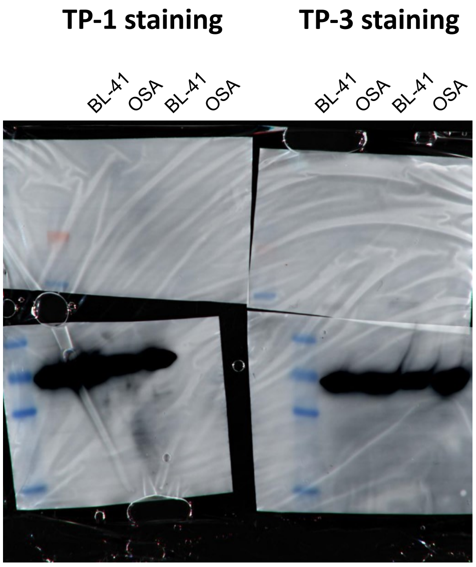

Supplementary methods 1. Western Blotting of BL-41 and OSA cells showing that TP-1 and TP-3 antibodies do not bind ALPL. The first column corresponds to the ladder, following by BL-41, OSA, BL-41, OSA (different lysate preparation or similar). Top gels correspond to TPs antibodies incubated and bottom gels correspond to B-actin staining.

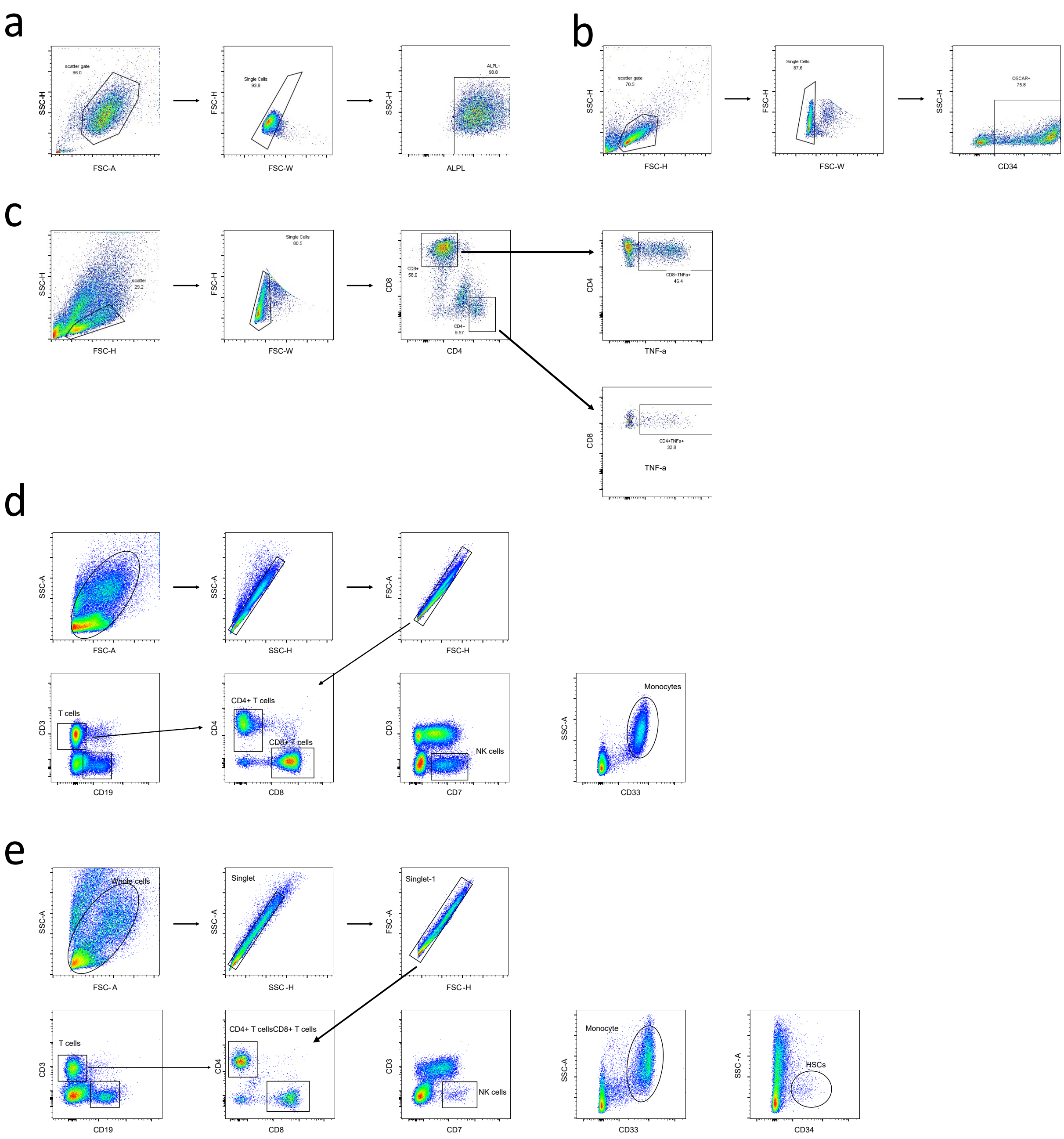

Supplementary methods 2. Gating strategy. **a**, Gating strategies used for TPs and ALPL detection in OS cell lines (data shown in Fig. 1a,b,d,f and Fig. 5c). Cells were gated based on size and granularity (FSC vs SSC). Singlets were gated by FSC-W vs FSC-H and TPs and ALPL were evaluated by the gating SSC vs ALPL/TPs. **b**, Gating strategies used to detect OSCAR+ T cells either by CD34 and/or aFab antibody staining (data shown in Fig. 2a). Cells were gated based on size and granularity (FSC vs SSC). Singlets were gated by FSC-W vs FSC-H and CD34 and/or aFab were evaluated by the gating SSC vs CD34 or SSC vs aFab. **c**, Gating strategies used to detect TNFa+ OSCAR CD8+ or CD4+ T cells (data shown in Fig. 5a, b). Cells were gated based on size and granularity (FSC vs SSC). Singlets were gated by FSC-W vs FSC-H. CD8+ or CD4+ T cells were evaluated by the gating CD4 vs CD8 and TNFa+ were obtained by the gating CD4 vs TNFa and CD8 vs TNFa. **d**, Gating strategies used TP3/ALPL detection in PBMCs (data shown in Fig. 5c). **e**, Gating strategies used TP3/ALPL detection in bone marrow, BM (data shown in Fig. 5c). For **d and e**, cells were gated based on size and granularity (FSC vs SSC). Singlets were gated by SSC-H vs SSC-A, and singlet-1 by FSC-H vs FSC-A. T cells and B cells were evaluated by the gating CD3 vs CD19. CD8+ or CD4+ T cells were evaluated by the gating CD4 vs CD8, NK cells were gating on CD7+ CD3-, Monocytes on CD33+ and Human Stem cells (HSCs) CD34+.
