# Supplementary material for: ALPL-1 is a target for chimeric antigen receptor therapy in osteosarcoma
Source: Nat Commun. 2023 Jun 8;14:3375. doi: 10.1038/s41467-023-39097-x (PMC10250459; doi:10.1038/s41467-023-39097-x)
Supplement: Supplementary file 3 — Description of Additional Supplementary Files [file 41467_2023_39097_MOESM3_ESM.pdf]

Description of Additional Supplementary Files

File Name: Supplementary Movie 1-3
Description: Movies of time-lapsed video showing Ca2+ increases in mock T (Data1), OSCAR-1 (Data 2) and OSCAR-3 (Data 3) cells after interaction with OSA tumor cells. OSA cells were seeded on Ibidi µ-slide the day before the experiment. CAR T cells loaded with Fura 2-AM were added before image recording. Images were acquired every 20 seconds for 80 cycles. CAR T cell Ca2+ level is color-coded, ranging from blue to red. Scale bar, 50 µm.
