# Supplementary material for: ALPL-1 is a target for chimeric antigen receptor therapy in osteosarcoma
Source: Nat Commun. 2023 Jun 8;14:3375. doi: 10.1038/s41467-023-39097-x (PMC10250459; doi:10.1038/s41467-023-39097-x)
Supplement: Supplementary file 7 — Reporting Summary [file 41467_2023_39097_MOESM7_ESM.pdf]

nature portfolio

Corresponding author(s): Sébastien Wälchli
Else Marit Inderberg

Last updated by author(s): 02/05/2023

Reporting Summary

Nature Portfolio wishes to improve the reproducibility of the work that we publish. This form provides structure for consistency and transparency in reporting. For further information on Nature Portfolio policies, see our Editorial Policies and the Editorial Policy Checklist.

Statistics

For all statistical analyses, confirm that the following items are present in the figure legend, table legend, main text, or Methods section.

n/a	Confirmed	
☐	☒	The exact sample size (*n*) for each experimental group/condition, given as a discrete number and unit of measurement
☐	☒	A statement on whether measurements were taken from distinct samples or whether the same sample was measured repeatedly
☐	☒	The statistical test(s) used AND whether they are one- or two-sided<br>*Only common tests should be described solely by name; describe more complex techniques in the Methods section.*
☐	☒	A description of all covariates tested
☐	☒	A description of any assumptions or corrections, such as tests of normality and adjustment for multiple comparisons
☐	☒	A full description of the statistical parameters including central tendency (e.g. means) or other basic estimates (e.g. regression coefficient) AND variation (e.g. standard deviation) or associated estimates of uncertainty (e.g. confidence intervals)
☐	☒	For null hypothesis testing, the test statistic (e.g. *F*, *t*, *r*) with confidence intervals, effect sizes, degrees of freedom and *P* value noted<br>*Give P values as exact values whenever suitable.*
☒	☐	For Bayesian analysis, information on the choice of priors and Markov chain Monte Carlo settings
☒	☐	For hierarchical and complex designs, identification of the appropriate level for tests and full reporting of outcomes
☒	☐	Estimates of effect sizes (e.g. Cohen's *d*, Pearson's *r*), indicating how they were calculated

Our web collection on statistics for biologists contains articles on many of the points above.

Software and code

Policy information about availability of computer code

Data collection	BD FACSDiva v8.0.1<br>IncuCyte S3 2019A<br>WorkOut v2.5<br>BioPlex Manager v6.1<br>MetaFluor Fluorescence Ratio Imaging<br>RTCA v2.1<br>G*Power v3.1.9.7
Data analysis	Flow cytometry data were analyzed with FlowJo v10.8.0<br>Figures with statistic were generated with GraphPad Prism v9<br>Image J v1.53e<br>Xenogen Spectrum system and Living Image version v3.2<br>For gene/transcription expression analysis the following softwares were used:<br>-Trim Galore v0.6.5, Cutadapt v2.10 and FastQC v0.11.9 (for FastQ file quality check) and MultiQC v1.9 (for summarizing)<br>- Kallisto v046.1 (for quantifying abundance of transcripts)<br>- Data were analyzed using R-CRAN v4.0 (Mac). The following R-packages were used: tximport v1.16.0 (import and summary of transcript-level abundance estimates), DESeq2 v1.28.1 Bioconductor 3.11 (expression gene/transcript analysis), edge v3.30.3 Bioconductor 3.11 (differential expression analysis of count data)

For manuscripts utilizing custom algorithms or software that are central to the research but not yet described in published literature, software must be made available to editors and reviewers. We strongly encourage code deposition in a community repository (e.g. GitHub). See the Nature Portfolio guidelines for submitting code & software for further information.

Data

Policy information about availability of data

All manuscripts must include a data availability statement. This statement should provide the following information, where applicable:

- Accession codes, unique identifiers, or web links for publicly available datasets
- A description of any restrictions on data availability
- For clinical datasets or third party data, please ensure that the statement adheres to our policy

The main data supporting the findings of the study are available within the paper and Supplementary Information file. Further data or material supporting the study are available from the corresponding author (S.W.) upon reasonable request. Data involving patient samples are not openly available for confidentiality reasons. ALPL-1 expression data FastQC files were downloaded from the European Nucleotide Archive, ENA (https://www.ebi.ac.uk/ena/browser/home). The reference transcriptome was Ensemble, GRCh38 (ftp://ftp.ensembl.org/pub/release100/fasta/homo_sapiens/cdna/Homo_sapiens.GRCh38.cdna.all.fa.gz). Source data are provided with this paper.

Human research participants

Policy information about studies involving human research participants and Sex and Gender in Research.

Reporting on sex and gender	No human research participants were involved. No sex and gender-based analyses have been performed because OS therapy will be administrated independently of the gender.
Population characteristics	No human research participants were recruited.
Recruitment	No human research participants were recruited.
Ethics oversight	Primary sarcoma tumors were collected at the Center for Orthopedic Innovations of the Mercy Miami Hospital (Florida, USA), according to regulations specified by the Nova Southeastern University Institutional Review Board (protocol # 2017-304).<br>OST-3 and OST-4 primary cell lines were generated from OS samples surgically resected at the Hospital Universitario Central de Asturias (Oviedo, Spain) after obtaining the approval of the Institutional Ethics Committee of the Principado de Asturias.<br>Use of BM and T cell material was approved by the Regional Committees for Medical Research Ethics South East Norway (approval 2013/624 and 2019/121).<br>Use of PBMC material was approved by the Barcelona Clinic Hospital Ethics Committee on institutional review board (IRB) (approval HCB/2018/0030). Use of BM sample material was approved by the Barcelona Clinic Hospital Ethics Committee (approval HCB/2017/1056).<br>Ethical committee of the Hospitales Universitaros Virgen Macarena y Virgen de Rocio (v.1-07/07/12/2021) and the Institutational Review Board of JCLRI approval (HCB/2017/1056).

Note that full information on the approval of the study protocol must also be provided in the manuscript.

Field-specific reporting

Please select the one below that is the best fit for your research. If you are not sure, read the appropriate sections before making your selection.

☒ Life sciences ☐ Behavioural & social sciences ☐ Ecological, evolutionary & environmental sciences

For a reference copy of the document with all sections, see nature.com/documents/nr-reporting-summary-flat.pdf

Life sciences study design

All studies must disclose on these points even when the disclosure is negative.

Sample size	No statistical methods were used to determine the sample size. CAR T cells generated from different healthy donors were used in separated experiments. It was reasoned that CAR T cells from 3 to 6 healthy donors would give valid account for donor variability based on our past experience with inter-donor variability. For in vivo study, a power analysis was conducted to determine the minimum sample size required to test the study hypothesis (G*Power v3.1.9.7). For in vitro experiments, each experiment was performed at least twice, when possible, as independent experiment (N=2), and with two to six technical replicates (n=2-6). The sample size was sufficiently powered to detect a difference between the group by statistical test.
Data exclusions	No data were excluded.
Replication	The experimental findings can be reliably reproduced.
Randomization	Mice were ear labeled and randomly assigned to an experimental group after tumour engraftment and prior to treatment with CAR T cells.
Blinding	No experiments were performed in blinded manner because the expertise for the readouts overlapped with the data collection.

Behavioural & social sciences study design

All studies must disclose on these points even when the disclosure is negative.

Study description	*Briefly describe the study type including whether data are quantitative, qualitative, or mixed-methods (e.g. qualitative cross-sectional, quantitative experimental, mixed-methods case study).*
Research sample	*State the research sample (e.g. Harvard university undergraduates, villagers in rural India) and provide relevant demographic information (e.g. age, sex) and indicate whether the sample is representative. Provide a rationale for the study sample chosen. For studies involving existing datasets, please describe the dataset and source.*
Sampling strategy	*Describe the sampling procedure (e.g. random, snowball, stratified, convenience). Describe the statistical methods that were used to predetermine sample size OR if no sample-size calculation was performed, describe how sample sizes were chosen and provide a rationale for why these sample sizes are sufficient. For qualitative data, please indicate whether data saturation was considered, and what criteria were used to decide that no further sampling was needed.*
Data collection	*Provide details about the data collection procedure, including the instruments or devices used to record the data (e.g. pen and paper, computer, eye tracker, video or audio equipment) whether anyone was present besides the participant(s) and the researcher, and whether the researcher was blind to experimental condition and/or the study hypothesis during data collection.*
Timing	*Indicate the start and stop dates of data collection. If there is a gap between collection periods, state the dates for each sample cohort.*
Data exclusions	*If no data were excluded from the analyses, state so OR if data were excluded, provide the exact number of exclusions and the rationale behind them, indicating whether exclusion criteria were pre-established.*
Non-participation	*State how many participants dropped out/declined participation and the reason(s) given OR provide response rate OR state that no participants dropped out/declined participation.*
Randomization	*If participants were not allocated into experimental groups, state so OR describe how participants were allocated to groups, and if allocation was not random, describe how covariates were controlled.*

Ecological, evolutionary & environmental sciences study design

All studies must disclose on these points even when the disclosure is negative.

Study description	*Briefly describe the study. For quantitative data include treatment factors and interactions, design structure (e.g. factorial, nested, hierarchical), nature and number of experimental units and replicates.*
Research sample	*Describe the research sample (e.g. a group of tagged Passer domesticus, all Stenocereus thurberi within Organ Pipe Cactus National Monument), and provide a rationale for the sample choice. When relevant, describe the organism taxa, source, sex, age range and any manipulations. State what population the sample is meant to represent when applicable. For studies involving existing datasets, describe the data and its source.*
Sampling strategy	*Note the sampling procedure. Describe the statistical methods that were used to predetermine sample size OR if no sample-size calculation was performed, describe how sample sizes were chosen and provide a rationale for why these sample sizes are sufficient.*
Data collection	*Describe the data collection procedure, including who recorded the data and how.*
Timing and spatial scale	*Indicate the start and stop dates of data collection, noting the frequency and periodicity of sampling and providing a rationale for these choices. If there is a gap between collection periods, state the dates for each sample cohort. Specify the spatial scale from which the data are taken*
Data exclusions	*If no data were excluded from the analyses, state so OR if data were excluded, describe the exclusions and the rationale behind them, indicating whether exclusion criteria were pre-established.*
Reproducibility	*Describe the measures taken to verify the reproducibility of experimental findings. For each experiment, note whether any attempts to repeat the experiment failed OR state that all attempts to repeat the experiment were successful.*
Randomization	*Describe how samples/organisms/participants were allocated into groups. If allocation was not random, describe how covariates were controlled. If this is not relevant to your study, explain why.*
Blinding	*Describe the extent of blinding used during data acquisition and analysis. If blinding was not possible, describe why OR explain why blinding was not relevant to your study.*

Did the study involve field work? ☐ Yes ☐ No

Field work, collection and transport

Field conditions	*Describe the study conditions for field work, providing relevant parameters (e.g. temperature, rainfall).*
Location	*State the location of the sampling or experiment, providing relevant parameters (e.g. latitude and longitude, elevation, water depth).*
Access & import/export	*Describe the efforts you have made to access habitats and to collect and import/export your samples in a responsible manner and in compliance with local, national and international laws, noting any permits that were obtained (give the name of the issuing authority, the date of issue, and any identifying information).*
Disturbance	*Describe any disturbance caused by the study and how it was minimized.*

Reporting for specific materials, systems and methods

We require information from authors about some types of materials, experimental systems and methods used in many studies. Here, indicate whether each material, system or method listed is relevant to your study. If you are not sure if a list item applies to your research, read the appropriate section before selecting a response.

Materials & experimental systems

n/a	Involved in the study	
☐	☒	Antibodies
☐	☒	Eukaryotic cell lines
☒	☐	Palaeontology and archaeology
☐	☒	Animals and other organisms
☒	☐	Clinical data
☒	☐	Dual use research of concern

Methods

n/a	Involved in the study	
☒	☐	ChIP-seq
☐	☒	Flow cytometry
☒	☐	MRI-based neuroimaging

Antibodies

Antibodies used

Flow cytometry:
anti-human CD4 (BV421; cloneOKT4; Biolegend; cat#317432; 2ul)
anti-human CD4 (BV605; clone RPA-T4; BD Biosciences; cat# 562658; 2ul)
anti-human CD8 (PE-Cy7; clone RPA-T8; Invitrogen; cat# 25-0088-42; 2ul)
anti-human TNFa (PE; clone Mab11; BD Pharmigen, BD Biosciences; cat#559321; 10ul)
mouse anti-human alkaline phosphatase (PE; clone B4-78; BD Pharmigen, BD Biosciences; cat# 561433; 3ul)
mouse anti-human alkaline phosphatase (PE; clone B4-78; R&D; cat# FAB1448P-100; 3ul)
TP-1, TP-3 antibodies from Hybridomas (our collection; dilution 1:200)
goat-anti mouse IgG (APC/PE; clone Poly4053; Biolegend; cat#405308, #405307; dilution 1:200)
goat- anti mouse IgG1 (PE; clone 11711; R&D; cat#IC002P; dilution 1:200)
anti-human CD34 (APC; clone 4H11; eBiosciences; cat#17-0349-42; 2.5ul)
anti-human CD45 (PE; clone HI30; BD biosciences; cat#560975; 2ul)
anti-human CD3 (BV605; clone OKT3; Invitrogen; cat#317322; 2ul)
anti-human CD3 (PerCP; clone SK7; BD Biosciences; cat#345766; dilution 1:100)
anti-human CD4 (BV421; clone RPA-T4; BD Horizon; cat#562424; dilution 1:100)
anti-human CD7 (FITC; clone M-T701; BD Pharmigen; cat#555360; dilution 1:100)
anti-human CD8 (BV510; clone SK1; BD Horizon; cat#563919; dilution 1:100)
anti-human CD19 (APC-H7; clone SJ25C1; BD Pharmigen; cat#550177; dilution 1.100)
anti-human CD33 (APC; clone WM53; BD Pharmigen; cat#551378; dilution 1:100)
anti-human CD34 (PE-Cy7; clone 8G12; BD Biosciences; cat#348791; dilution 1:100)
goat anti-mouse IgG F(ab')2 (AF-647; Cell signaling; cat#4410; dilution 1:5)
anti-mouse Fab biotinilated (Jackson ImmunoResearch; cat#B0529; dilution 1:200)
streptavidin (PE; BD Biosciences; cat#554061; dilution 1:400)

Western Blot:
human alkaline phosphatase (ALPL) (clone 928929; R&D; cat#MAB29092; 0.25ug/ml)
IGSF-1 polyclonal (Thermofisher; cat#PA5-42088; 1:1000)
Beta actin monoclonal (clone 15G5A11/E2; Thermofisher; cat#MA1-140; 1:2000)
goat anti-mouse IgG (H+L) horseradish peroxidase (HRP) secondary antibody (polyclonal; Thermo Fisher Scientific; cat#62-6520; dilution of 1:2500)
goat anti-rabbit IgG (H+L) horseradish peroxidase (HRP) secondary antibody (polyclonal; Thermo Fisher Scientific; cat# 31460; dilution of 1:2000)

IHC:

BOND Polymer Refine Detection (Leica Biosystem; cat#DS9800)

CTV-based proliferation assay:
CellTrace™ Violet Cell Proliferation Kit, for flow cytometry (Invitrogen, cat#C34571). Final concentration of 2.5 µM
7-Aminoactinomycin D (7-AAD) dye (BD Via-Probe, BD Biosciences; cat# 559925;100ul/test)

Validation

TP-1 and TP-3 antibodies were previously validated and published as Osteosarcoma specific antibodies. Immunohistochemical studies on frozen tissue sections showed that TP-1 and TP-3 antibodies bound to TPX cells and to cells from another osteosarcoma, but not to autologous skin fibroblasts. Binding of TP-I (TP-3) was demonstrated to 15/15 (15/15) osteosarcomas, 3/3 (2/2) synovial sarcomas, 7/9 (6/8) malignant fibrous histiocytomas, 2/2 (1/1) malignant hemangiopericytomas, 1/2 (1/2) chondrosarcomas and 3/6 (1/3) unclassified sarcomas. They did not bind to sections of 66 different non-sarcomatous malignancies, or to any of a range of normal adult and fetal tissues, although some weak staining of proximal kidney tubules was seen (Bruland OS, et al; Int. J. Cancer. 1986; Bruland OS, et al; Cancer Res. 1988). TP-1 was tested for clinical immunoscintigraphy in a pilot study involving 5 patients with bone sarcomas for the ability to detect lung metastases (Bruland OS, et al; European Journal of Cancer. 1994). TP-3 antibody fragment was labeled using the N-succinimidyl 8-[(4'-[18F]fluorobenzyl)amino]suberate acylation agent. 18F-labeled Fab fragment of TP-3 was used as PET imaging agent in four dogs with histologically confirmed osteogenic sarcoma (Page RL, et al; J Nucl Med. 1994). TP-1 and TP-3 antibody specificity for OS cells was confirmed in this study by flow cytometry-based assay and IHC on OS tumour cell lines, non-OS tumour cell lines, primary or cell lines from normal tissues.

Commercial primary antibodies used in this study in flow cytometry were validated by the manufacturer (Biolegend, BD Biosciences, Invitrogen, BD Pharmingen, R&D, eBiosciences, Cell Signaling, Jackson Immunoresearch), and the antibody concentrations were gathered from vendors datasheets.

https://www.biolegend.com/en-gb/clone-search/brilliant-violet-421-anti-human-cd4-antibody-7775
https://www.bdbiosciences.com/en-nz/products/reagents/flow-cytometry-reagents/research-reagents/single-color-antibodies-ruo/bv605-mouse-anti-human-cd4.562658
https://www.thermofisher.com/antibody/product/CD8a-Antibody-clone-RPA-T8-Monoclonal/25-0088-42
https://www.bdbiosciences.com/en-au/products/reagents/flow-cytometry-reagents/research-reagents/single-color-antibodies-ruo/pe-mouse-anti-human-tnf.559321
https://www.bdbiosciences.com/en-us/products/reagents/flow-cytometry-reagents/research-reagents/single-color-antibodies-ruo/pe-mouse-anti-human-alkaline-phosphatase.561433
https://www.rndsystems.com/products/human-alkaline-phosphatase-alpl-pe-conjugated-antibody-b4-78_fab1448p
https://www.biolegend.com/fr-lu/products/apc-goat-anti-mouse-igg-minimal-x-reactivity-1383
https://www.rndsystems.com/products/mouse-igg-1-pe-conjugated-antibody_ic002p
https://www.thermofisher.com/antibody/product/CD34-Antibody-clone-4H11-Monoclonal/17-0349-42
https://www.bdbiosciences.com/en-au/products/reagents/flow-cytometry-reagents/research-reagents/single-color-antibodies-ruo/pe-mouse-anti-human-cd45.560975
https://www.biolegend.com/en-us/products/brilliant-violet-605-anti-human-cd3-antibody-7666?GroupID=BLG4203
https://www.bdbiosciences.com/en-eu/products/reagents/flow-cytometry-reagents/clinical-diagnostics/single-color-antibodies-asr-ivd-ce-ivd/cd3-percp.345766
chrome-extension://efaidnbmnnnibpcajpcglclefindmkaj/https://www.bdbiosciences.com/content/dam/bdb/products/global/reagents/flow-cytometry-reagents/research-reagents/single-color-antibodies-ruo/562424_base/pdf/562424.pdf
https://www.bdbiosciences.com/en-sg/products/reagents/flow-cytometry-reagents/research-reagents/single-color-antibodies-ruo/fitc-mouse-anti-human-cd7.555360
https://www.bdbiosciences.com/en-us/products/reagents/flow-cytometry-reagents/research-reagents/single-color-antibodies-ruo/bv510-mouse-anti-human-cd8.563919
https://www.bdbiosciences.com/en-us/products/reagents/flow-cytometry-reagents/research-reagents/single-color-antibodies-ruo/apc-h7-mouse-anti-human-cd19.560177
https://www.bdbiosciences.com/en-us/products/reagents/flow-cytometry-reagents/research-reagents/single-color-antibodies-ruo/apc-mouse-anti-human-cd33.551378
https://www.bdbiosciences.com/en-us/products/reagents/flow-cytometry-reagents/clinical-discovery-research/single-color-antibodies-ruo-gmp/pe-cy-7-mouse-anti-human-cd34.348791
https://www.cellsignal.com/products/secondary-antibodies/anti-mouse-igg-h-l-f-ab-2-fragment-alexa-fluor-647-conjugate/4410

Commercial primary antibodies used in this study for Western Blotting were validated by the manufacturer as reported in the antibody data sheet. Human Alkaline Phosphatase/ALPL Antibody (Clone # 928929; R&D, Catalog Number MAB29092) was validated for WB on lysates of HeLa human cervical epithelial carcinoma cell line, BG01V human embryonic stem cells, and NTera-2 human testicular embryonic carcinoma cell line. IGSF-1 antibody (polyclonal; Thermofisher, Catalog Number PA5-42088) was validated for WB in human HepG2 cell lysate.

Eukaryotic cell lines

Policy information about cell lines and Sex and Gender in Research

Cell line source(s)

The OS cell lines OSA, U2OS, G-292, 143B, MG-63 and SaOS-2, the Burkitt Lymphoma cell line BL-41, the human liver cancer cell line HepG2, fibroblast MRC-5 cell line, and the endothelial Hulec-5a cell line were obtained from the American Type Culture Collection (ATCC).
OHS cell line was established at the Norwegian Radium Hospital as referred in Fodstad O, et al; International Journal of Cancer. 1986 (reference n.27 in the paper).
The human metastatic OS LM7 cell line was a kind gift from Dr. Eugenie Kleinerman (The University of Texas MD Anderson Cancer Center).

	Human embryonic kidney cell line HEK-293 was from our collection, originally from cell Biolabs Inc..<br>The OST-3 and OST-4 primary cell lines were generated from OS samples surgically resected at the Hospital Universitario Central de Asturias (Oviedo, Spain) after obtaining the approval of the Institutional Ethics Committee of the Principado de Asturias.<br>Primary sarcoma tumors were collected at the Center for Orthopedic Innovations of the Mercy Miami Hospital (Florida, USA), (protocol #2017-304).<br>Primary human renal epithelial cells (HREpC) were purchased from PromoCell (Promocell).<br>Primary human hepatocytes (HH) and primary human pulmonary alveolar epithelial cells (HPAEpiC) were purchased from ScienCell Research Laboratories (ScienCell Research Laboratories).<br>Human primary mesenchymal cells (MSCs) were isolated from healthy donor BM and used to differentiate into Osteoblasts.
Authentication	Authentication and associated testing was performed by the vendors (ATCC, Cell Biolabs Inc., Promocell, ScienCell Research Laboratories). Some of the cell lines, OSA and the LM-7 (SaOS-2 derivative) were sent for authentication to Labcorp (Burlington, NC, US).
Mycoplasma contamination	Cell lines were routinely tested at our unit for Mycoplasma contamination (every 3-6 months). (VenorGeM Cells were mycoplasma free at the time of experiment.
Commonly misidentified lines<br>(See ICLAC register)	No commonly misidentified cell lines were used.

Palaeontology and Archaeology

Specimen provenance	*Provide provenance information for specimens and describe permits that were obtained for the work (including the name of the issuing authority, the date of issue, and any identifying information). Permits should encompass collection and, where applicable, export.*
Specimen deposition	*Indicate where the specimens have been deposited to permit free access by other researchers.*
Dating methods	*If new dates are provided, describe how they were obtained (e.g. collection, storage, sample pretreatment and measurement), where they were obtained (i.e. lab name), the calibration program and the protocol for quality assurance OR state that no new dates are provided.*

☐ Tick this box to confirm that the raw and calibrated dates are available in the paper or in Supplementary Information.

Ethics oversight	*Identify the organization(s) that approved or provided guidance on the study protocol, OR state that no ethical approval or guidance was required and explain why not.*

Note that full information on the approval of the study protocol must also be provided in the manuscript.

Animals and other research organisms

Policy information about studies involving animals; ARRIVE guidelines recommended for reporting animal research, and Sex and Gender in Research

Laboratory animals	Non-obese diabetic (NOD).Cg-Prkdcscid Il2rgtm1Wjl/SzJ (NSG) mice, female, between six-to-eight-weeks-old or between six-to-ten-weeks-old were included in the study groups. Mice were housed in 12 hour light-dark cycles with humidity between 30-70% at ambient temperature of 20-26 degrees Celsius. The study and control animals were housed in the same room. The animal facilities are specific pathogen-free facility. Euthanasia was conducted via exsanguination under isoflurane anesthesia followed by cervical dislocation or by cervical dislocation only.
Wild animals	The study did not involved wild animals.
Reporting on sex	All animals were female
Field-collected samples	The study did not involved field-collected samples.
Ethics oversight	Mouse received humane care in compliance with the Norwegian Food Safety Authority (approval ID11118) and the Spanish ethics committee on animal experimentation and welfare (FGA-17-0030).

Note that full information on the approval of the study protocol must also be provided in the manuscript.

Clinical data

Policy information about clinical studies
All manuscripts should comply with the ICMJE guidelines for publication of clinical research and a completed CONSORT checklist must be included with all submissions.

Clinical trial registration	*Provide the trial registration number from ClinicalTrials.gov or an equivalent agency.*
Study protocol	*Note where the full trial protocol can be accessed OR if not available, explain why.*

Data collection | Describe the settings and locales of data collection, noting the time periods of recruitment and data collection.

Outcomes | Describe how you pre-defined primary and secondary outcome measures and how you assessed these measures.

Dual use research of concern

Policy information about dual use research of concern

Hazards

Could the accidental, deliberate or reckless misuse of agents or technologies generated in the work, or the application of information presented in the manuscript, pose a threat to:

No	Yes	
☒	☐	Public health
☒	☐	National security
☒	☐	Crops and/or livestock
☒	☐	Ecosystems
☒	☐	Any other significant area

Experiments of concern

Does the work involve any of these experiments of concern:

No	Yes	
☒	☐	Demonstrate how to render a vaccine ineffective
☒	☐	Confer resistance to therapeutically useful antibiotics or antiviral agents
☒	☐	Enhance the virulence of a pathogen or render a nonpathogen virulent
☒	☐	Increase transmissibility of a pathogen
☒	☐	Alter the host range of a pathogen
☒	☐	Enable evasion of diagnostic/detection modalities
☒	☐	Enable the weaponization of a biological agent or toxin
☒	☐	Any other potentially harmful combination of experiments and agents

ChIP-seq

Data deposition

☐ Confirm that both raw and final processed data have been deposited in a public database such as GEO.

☐ Confirm that you have deposited or provided access to graph files (e.g. BED files) for the called peaks.

Data access links
May remain private before publication. | *For "Initial submission" or "Revised version" documents, provide reviewer access links. For your "Final submission" document, provide a link to the deposited data.*

Files in database submission | *Provide a list of all files available in the database submission.*

Genome browser session
(e.g. UCSC) | *Provide a link to an anonymized genome browser session for "Initial submission" and "Revised version" documents only, to enable peer review. Write "no longer applicable" for "Final submission" documents.*

Methodology

Replicates | *Describe the experimental replicates, specifying number, type and replicate agreement.*

Sequencing depth | *Describe the sequencing depth for each experiment, providing the total number of reads, uniquely mapped reads, length of reads and whether they were paired- or single-end.*

Antibodies | *Describe the antibodies used for the ChIP-seq experiments; as applicable, provide supplier name, catalog number, clone name, and lot number.*

Peak calling parameters | *Specify the command line program and parameters used for read mapping and peak calling, including the ChIP, control and index files used.*

Data quality | *Describe the methods used to ensure data quality in full detail, including how many peaks are at FDR 5% and above 5-fold enrichment.*

Software

Describe the software used to collect and analyze the ChIP-seq data. For custom code that has been deposited into a community repository, provide accession details.

Flow Cytometry

Plots

Confirm that:

☒ The axis labels state the marker and fluorochrome used (e.g. CD4-FITC).

☒ The axis scales are clearly visible. Include numbers along axes only for bottom left plot of group (a 'group' is an analysis of identical markers).

☒ All plots are contour plots with outliers or pseudocolor plots.

☒ A numerical value for number of cells or percentage (with statistics) is provided.

Methodology

Sample preparation

Extracellular staining:
Single-cell suspensions were stained with fluorescent antibodies in PBS supplemented with 2% of fetal bovine serum (FBS) for 15 minutes at room temperature in the dark. Then, sample were washed in PBS with 2% FBS and resuspended in the same media.
For indirect staining, the primary antibody labeling was followed by staining with a compatible fluorescent dye-conjugated secondary antibody.

Intracellular staining:
For TNFα production, CAR T cells were co-cultured with tumor cells (Osteosarcoma and non osteosarcoma cells) in the presence of BD GolgiPlug and BD Golgistop for 6h. Subsequently, cells were harvested and stained extracellularly and intracellularly using the PerFix-nc kit according to the manufacturer's instructions (Beckman Coulter).

CTV proliferation assay:
Initial FSC-H/SSC-H gates were drawn on the cell populations, excluding debris, followed by SSC-A/SSC-W gate to define single cells. A further gate SSC-H/GFP negative was drawn to exclude possible leftover debris from dead ALPL-1 positive target cells which were GFP positive.
Boundary between positive and negative CTV cells was established using cells not labelled for CTV.

Instrument

Cells were acquired on FACS Aria (BD Biosciences) or Sony SH800 Cell Sorters (Sony Biotechnology Inc.) were used for cell sorting.

Software

BD FACSDiva v8.0.1 and FlowJo v10.8.0 were used to collect and analyze the flow cytometry data, respectively.

Cell population abundance

The sorted tumor cell lines were analyzed for purity.

Gating strategy

All experiments were performed using appropriate controls. Before gating on fluorescences, single cells were identified by using the following general gate strategy:
1) "FSC vs SSC" dot plot to draw a scatter gate
2) "FSC-W vs FSC-H" dot plot to draw singlet gate
3) "SSC vs investigated antibody" dot plot to draw on antibody positive cells

In details,
ALPL, TP-1 and TP-3 expression was visualized in
1) "FSC vs SSC" dot plot to draw a scatter gate
2) "FSC-W vs FSC-H" dot plot to draw singlet gate
3) " SSC vs ALPL/TPs" dot plot to identify ALP/TP positive cells, as reported in Supplementary Information file.

CAR expressing T cells were identified by:
1) "FSC vs SSC" dot plot (scatter gate)
2) "FSC-W vs FSC-H" dot plot (singlet gate)
3) " SSC vs CD34" or "SSC vs aFab" dot plot (SSC/CD34+ or SSC/aFab+), as reported in Supplementary Information file.

For TNFa detection single cells were gated using :
1) "FSC vs SSC" dot plot (scatter gate)
2) "FSC-W vs FSC-H"dot plot (singlet gate)
3) "CD4 vs CD8" dot plot to identify CD8+ or CD4+ T cells (CD8 gate and CD4 gate)
4) "CD4 vs TNFa" dot plot (CD4+/TNFa+) and " CD8 vs TNFa" dot plot (CD8+/TNFa+), as reported in Supplementary Information file.

PBMCs, BM cell population were identified by the following gate strategy:

1) "FSC vs SSC" dot plot (whole cells)
2) "SSC-H vs SSC-A" dot plot (Singlet)
3) "FSC-H vs FSC-A" dot plot (Singlet I)

- T cells CD3+ --> CD4+ or CD8`+ T cells.
- B cells CD19+.
- NK cells CD7+ CD3-
- Monocytes CD33+
- Human Stem cells (HSCs) CD34+

☒ Tick this box to confirm that a figure exemplifying the gating strategy is provided in the Supplementary Information.

Magnetic resonance imaging

Experimental design

Design type	*Indicate task or resting state; event-related or block design.*
Design specifications	*Specify the number of blocks, trials or experimental units per session and/or subject, and specify the length of each trial or block (if trials are blocked) and interval between trials.*
Behavioral performance measures	*State number and/or type of variables recorded (e.g. correct button press, response time) and what statistics were used to establish that the subjects were performing the task as expected (e.g. mean, range, and/or standard deviation across subjects).*

Acquisition

Imaging type(s)	*Specify: functional, structural, diffusion, perfusion.*
Field strength	*Specify in Tesla*
Sequence & imaging parameters	*Specify the pulse sequence type (gradient echo, spin echo, etc.), imaging type (EPI, spiral, etc.), field of view, matrix size, slice thickness, orientation and TE/TR/flip angle.*
Area of acquisition	*State whether a whole brain scan was used OR define the area of acquisition, describing how the region was determined.*

Diffusion MRI ☐ Used ☐ Not used

Preprocessing

Preprocessing software	*Provide detail on software version and revision number and on specific parameters (model/functions, brain extraction, segmentation, smoothing kernel size, etc.).*
Normalization	*If data were normalized/standardized, describe the approach(es): specify linear or non-linear and define image types used for transformation OR indicate that data were not normalized and explain rationale for lack of normalization.*
Normalization template	*Describe the template used for normalization/transformation, specifying subject space or group standardized space (e.g. original Talairach, MNI305, ICBM152) OR indicate that the data were not normalized.*
Noise and artifact removal	*Describe your procedure(s) for artifact and structured noise removal, specifying motion parameters, tissue signals and physiological signals (heart rate, respiration).*
Volume censoring	*Define your software and/or method and criteria for volume censoring, and state the extent of such censoring.*

Statistical modeling & inference

Model type and settings	*Specify type (mass univariate, multivariate, RSA, predictive, etc.) and describe essential details of the model at the first and second levels (e.g. fixed, random or mixed effects; drift or auto-correlation).*
Effect(s) tested	*Define precise effect in terms of the task or stimulus conditions instead of psychological concepts and indicate whether ANOVA or factorial designs were used.*

Specify type of analysis: ☐ Whole brain ☐ ROI-based ☐ Both

Statistic type for inference (See Eklund et al. 2016)	*Specify voxel-wise or cluster-wise and report all relevant parameters for cluster-wise methods.*
Correction	*Describe the type of correction and how it is obtained for multiple comparisons (e.g. FWE, FDR, permutation or Monte Carlo).*

Models & analysis

n/a	Involved in the study
☐	☐ Functional and/or effective connectivity
☐	☐ Graph analysis
☐	☐ Multivariate modeling or predictive analysis

Functional and/or effective connectivity	*Report the measures of dependence used and the model details (e.g. Pearson correlation, partial correlation, mutual information).*
Graph analysis	*Report the dependent variable and connectivity measure, specifying weighted graph or binarized graph, subject- or group-level, and the global and/or node summaries used (e.g. clustering coefficient, efficiency, etc.).*
Multivariate modeling and predictive analysis	*Specify independent variables, features extraction and dimension reduction, model, training and evaluation metrics.*
